# Influence of nitrate and nitrite concentration on N_2_O production via dissimilatory nitrate/nitrite reduction to ammonium in *Bacillus paralicheniformis* LMG 6934

**DOI:** 10.1002/mbo3.592

**Published:** 2018-03-05

**Authors:** Yihua Sun, Paul De Vos, Anne Willems

**Affiliations:** ^1^ Laboratory of Microbiology Department of Biochemistry and Microbiology Ghent University Gent Belgium

**Keywords:** ammonification, dissimilatory nitrate/nitrite reduction to ammonium, nitrate respiration, nitrogen assimilation

## Abstract

Until now, the exact mechanisms for N_2_O production in dissimilatory nitrate/nitrite reduction to ammonium (DNRA) remain underexplored. Previously, we investigated this mechanism in *Bacillus licheniformis* and *Bacillus paralicheniformis*, ubiquitous gram‐positive bacteria with many industrial applications, and observed significant strain dependency and media dependency in N_2_O production which was thought to correlate with high residual NO_2_^−^. Here, we further studied the influence of several physicochemical factors on NO_3_^−^ (or NO_2_^−^) partitioning and N_2_O production in DNRA to shed light on the possible mechanisms of N_2_O production. The effects of NO_3_^−^ concentrations under variable or fixed C/N‐NO_3_^−^ ratios, NO_2_^−^ concentrations under variable or fixed C/N‐NO_2_^−^ ratios, and NH_4_^+^ concentrations under fixed C/N‐NO_3_^−^ ratios were tested during anaerobic incubation of soil bacterium *B. paralicheniformis* LMG 6934 (previously known as *B*. *licheniform*is), a strain with a high nitrite reduction capacity. Monitoring of growth, NO_3_^−^, NO_2_^−^, NH_4_^+^ concentration, and N_2_O production in physiological tests revealed that NO_3_^−^ as well as NO_2_^−^ concentration showed a linear correlation with N_2_O production. Increased NO_3_^−^ concentration under fixed C/N‐NO_3_^−^ ratios, NO_2_^−^ concentration, and NH_4_^+^ concentration had a significant positive effect on NO_3_^−^ (or NO_2_^−^) partitioning ([N–NH_4_^+^]/[N–N_2_O]) toward N_2_O, which may be a consequence of the (transient) accumulation and subsequent detoxification of NO_2_^−^. These findings extend the information on several physiological parameters affecting DNRA and provide a basis for further study on N_2_O production during this process.

## 1 INTRODUCTION

Nowadays, there is an increasing concern about the year‐by‐year rising emissions of N_2_O from soil, as it is a potent greenhouse gas that damages the ozone layer (Daniel et al., 2007; Solomon et al., 2007; Wuebbles, 2009). Denitrification has been considered as the dominant NO_3_^−^ reducing process in soil, in which NO_3_^−^ is sequentially converted to NO_2_^−^, NO, N_2_O, and N_2_. However, recently, field surveys (Bu et al., 2017; Silver, Herman, & Firestone, 2001; Silver, Thompson, Reich, Ewel, & Firestone, 2005; Song, Lisa, & Tobias, 2014; Yin et al., 2017) and research with pure cultures (Bleakley & Tiedje, 1982; Mania, Heylen, Spanning, & Frostegård, 2014; Smith & Zimmerman, 1981; Stremińska, Felgate, Rowley, Richardson, & Baggs, 2012; Sun, De Vos, & Heylen, 2016) have suggested that NO_3_^−^‐ammonifying bacteria could be a significant source of N_2_O. Ammonification or dissimilatory NO_3_^−^ reduction to NH_4_^+^ (DNRA) is the reduction in NO_3_^−^ to NH_4_^+^, via NO_2_^−^ (Cole, 1996; Simon, 2002), with the concomitant production of nonstoichiometric amounts of N_2_O amounting to around 3%–36% of consumed NO_3_^−^ (Bleakley & Tiedje, 1982). DNRA can follow different scenarios, with respiratory membrane‐bound NarG, cytoplasmic NasBC, or periplasmic NO_3_^−^ reductase NapA for NO_3_^−^ reduction to NO_2_^−^, followed by NO_2_^−^ reduction to NH_4_^+^ via cytoplasmic nitrite reductase NirB or a periplasmic nitrite reductase NrfA (Bothe, Ferguson, & Newton, 2006), with NirB induced under high NO_3_^−^ concentration and NrfA induced by low NO_3_^−^ concentration (Wang & Gunsalus, 2000). The exact mechanisms for N_2_O production remain underexplored. They may differ between ammonifiers and most likely depend on the enzymes involved in the DNRA process. In *Escherichia coli* K‐12, NO was shown to be produced by NrfA under the regulation of Fnr and mutants lacking Hmp, NarG or Fnr did not produce NO (Corker & Poole, 2003). In *Salmonella enterica* serovar *Typhimurium*, NarGHI was responsible for NO generation from NO_2_^−^ (Gilberthorpe & Poole, 2008). The produced NO in these two bacteria will be reduced to N_2_O by flavohemoglobin Hmp and the di‐iron‐centered flavorubredoxin NorV with its NADH‐dependent oxidoreductase NorW. Hmp is phylogenetically widespread in both denitrifying bacteria and nondenitrifiers. It can oxidize NO to NO_3_^−^ in the presence of oxygen and reduce NO to N_2_O under anoxic conditions (Kim, Orii, Lloyd, Hughes, & Poole, 1999). However, not Hmp but NorVW (Gomes et al., 2002) may be the significant source of N_2_O, which can detoxify NO under micro‐oxic or anaerobic conditions (Torres et al., 2016). Besides, canonical NO reductase—Nor, which mostly exists in denitrifiers, was also found in certain DNRA bacteria. For instance, *Bacillus vireti* LMG 21834^T^ performs DNRA by NarG, NrfA, and Nor (CbaA), with additional NosZ partially reducing N_2_O to N_2_ (Mania, Heylen, Spanning, & Frostegård, 2016; Mania et al., 2014). Similarly, *Bacillus paralicheniform*is LMG 6934, LMG7559 (renamed since 2015 (Dunlap, Kwon, Rooney, & Kim, 2015)), and *Bacillus licheniform*is LMG17339 possess NarG, NirBD, and Nor, but not NosZ (Sun et al., 2016). While, the mutants of *Salmonella typhimurium Typhimurium* lacking Hmp, NorV, and NrfA and of *E.coli* lacking NirB, NrfA, NorV, and Hmp still can reduce NO, suggesting that there are other mechanisms of NO reduction uncharacterized (Mills, Rowley, Spiro, Hinton, & Richardson, 2008).

As denitrification and DNRA are the two well‐known NO_3_^−^‐consuming pathways in soil, with the former contributing to nitrogen loss to the atmosphere and the latter mainly leading to nitrogen retention in soil, studies with respect to different factors influencing these two pathways have been widely performed. It is well known that DNRA is favored over denitrification at higher C/N‐NO_3_^−^ ratios or NO_3_^−^ limitation (Van den Berg, Van Dongen, Abbas, & Van Loosdrecht, 2015; Yoon, Cruz‐Garcia, Sanford, Ritalahti, & Löffler, 2015), higher pH (Schmidt, Richardson, & Baggs, 2011; Yoon, Cruz‐Garcia, et al., 2015), higher temperature (Ogilvie, Rutter, & Nedwell, 1997; Yoon, Sanford, & Loeffler, 2015), and certain NO_2_^−^ to NO_3_^−^ ratios (Schmidt et al., 2011; Yoon, Sanford, et al., 2015). However, the influence of these environmental drivers on NO_3_^−^ partitioning to NH_4_^+^ and N_2_O in DNRA remains underexplored, although increased understanding might help unravel the underlying mechanisms and regulation of N_2_O production. Early work by Smith showed that higher C/NO_3_^−^ ratios under constant or decreasing NO_3_^−^ concentration (Smith, 1981) favored NO_3_^−^ partitioning to N_2_O in *Citrobacter* sp. with glucose as energy source and suggested that N_2_O production was induced by (transient) accumulation of NO_2_^−^. However, recently, it was found, both in batch and continuous cultures of *Citrobacter* sp. and *Bacillus* sp., that low C/N‐NO_3_^−^ (C limitation, N sufficiency) ratios resulted in higher NO_2_^−^ accumulation accompanied by higher N_2_O production compared to high C/N‐NO_3_^−^ with constant initial glycerol concentration as carbon source and variable NO_3_^−^ concentration (Stremińska et al., 2012).

It has been generally known that NH_4_^+^ inhibits assimilatory NO_3_^−^ reduction (general N control) (Schreier, Brown, Hirschi, Nomellini, & Sonenshein, 1989; Stouthamer, 1976), increases growth rate of cells (Sun, De Vos, & Willems, 2017), and does not repress dissimilatory NO_3_^−^ reduction (Konohana, Murakami, Nanmori, Aoki, & Shinke, 1993). In *B. licheniformis*, NO_3_^−^ reductase activity increased with rising initial concentrations of NH_4_^+^, but with an upper limit of 46 mmol/L, suggesting that the activity is not for NO_3_^−^ assimilation but for other physiological functions containing a dissimilatory NO_3_^−^ reduction (Konohana et al., 1993). However, no previous work has been performed on the influence of NH_4_^+^ on N_2_O production in DNRA. As NH_4_^+^ can react with multiple nitrogen regulation sensors (TnrA, CodY, and GlnR) and the mechanism of N_2_O production and regulation of nitrogen metabolism are underexplored in DNRA strains, it is possible that NH_4_^+^ can influence NO_3_^−^ partitioning to N_2_O.

*B. (para)licheniformis* is a spore‐forming gram‐positive bacterium that can be isolated from soils and plant material all over the world but was never reported to be pathogenic for either animals or plants (Sneath, Mair, Sharpe, & Holt, 1986). In our previous study, we investigated three strains of *B. (para)licheniform*is (as mentioned above) which were disguised as denitrifiers and proved that they are N_2_O emitters performing DNRA probably by expression of *narG*,* nirB*,* qNor,* and *hmp*, with up to one‐third of all NO_3_^−^ converted to N_2_O (Sun et al., 2016). They are therefore suitable model organisms to study the mechanism of N_2_O production during DNRA and to supplement the insights of environmental drivers influencing DNRA. Following our observation of N_2_O production being correlated to high residual NO_2_^−^, here we used the soil bacterium *B. paralicheniformis* LMG 6934, selected for its high nitrite tolerance and efficient nitrite reduction ability, to study in detail the influence of NO_3_^−^, NO_2_^−^, and NH_4_^+^ concentrations on N_2_O production via DNRA in batch cultures.

## 2 MATERIALS AND METHODS

### 2.1 Strains

*Bacillus paralicheniformis* LMG 6934 was obtained from the BCCM/LMG bacteria collection. It was grown aerobically at 37°C on TSA for 2 days, followed by two subcultivations on TSA before use in growth experiments in mineral media.

### 2.2 Growth experiments

Anaerobic growth experiments were performed in mineral medium (containing 4.6 mmol/L NH_4_^+^) supplemented with 10 mmol/L potassium NO_3_^−^ as electron acceptor and 30 mmol/L glucose as electron donor unless stated otherwise. Mineral medium was as described by Stanier, Palleroni, and Doudoroff (1966), including 10 mmol/L phosphate buffer (pH 6.92 ± 0.05), 2.3 mmol/L (NH_4_)_2_SO_4_, 0.4 mmol/L MgSO_4_·7H_2_O, 0.04 mmol/L CaCl_2_·2H_2_O, 27 μmol/L EDTA, 25 μmol/L FeSO_4_·7H_2_O, 10 μmol/L ZnSO_4_·7H_2_O, 25 μmol/L MnSO_4_·H_2_O, 3.8 μmol/L CuSO_4_·5H_2_O, 2 μmol/L Co(NO_3_)_2_·6H_2_O, and 0.196 μmol/L (NH_4_)_6_Mo_7_O_24_·24H_2_O. Serum vials (120 ml) were soaked in 1 mol/L HCl overnight to remove growth inhibiting substances and subsequently washed five times with distilled water before use. Serum vials with 50 ml medium were sealed with black butyl rubber stoppers. After autoclaving, the headspace of the serum vials was replaced via five cycles of evacuating and refilling with helium. Serum vials were inoculated (1% v/v) with a bacterial suspension of OD_600_ of 1.0 ± 0.05. Each growth experiment was performed in triplicate, and noninoculated media in duplicate were included to check for potential nitrosation reactions in sterile medium, which were proved negligible after measurement. After inoculation, serum vials were incubated at 37°C, 150 rpm, for 72 hr for endpoint analysis or for 192 hr for detailed growth experiments. Gas samples and culture samples were taken at the start and the end of the experiment, or at various time points over the incubation for detailed analysis (see below).

Mineral media with different supplements were designed and tested to study the effect of several factors on NO_3_^−^ partitioning to NH_4_^+^ and N_2_O: (1) different NO_3_^−^ concentrations (5 mmol/L, 10 mmol/L, and 15 mmol/L) and 30 mmol/L glucose resulting in variable C/N‐NO_3_^−^ ratios of 36, 18, and 12; (2) different NO_3_^−^ concentrations (5 mmol/L, 10 mmol/L, and 15 mmol/L) under identical C/N‐NO_3_^−^ ratio of 12 (glucose 10 mmol/L, 20 mmol/L, and 30 mmol/L, respectively); (3) different NO_2_^−^ concentrations without NO_3_^−^ (1 mmol/L, 5 mmol/L, and 10 mmol/L) and 30 mmol/L glucose resulting in variable C/N‐NO_2_^−^ ratios of 180, 36, and 18; (4) different NO_2_^−^ concentrations (1 mmol/L, 5 mmol/L, and 10 mmol/L) under identical C/N‐NO_2_^−^ ratio of 18 (glucose 3 mmol/L, 15 mmol/L, and 30 mmol/L, respectively); (5) different NH_4_^+^ concentrations (0 mmol/L, 1 mmol/L, 4.6 mmol/L, and 10 mmol/L) and 10 mmol/L NO_3_^−^, 30 mmol/L glucose, resulting a C/N‐NO_3_^−^ ratio of 18. Under all conditions, incubation was limited to 72 hr for endpoint analysis. However, in addition, in setup (4), serum vials were also incubated for a longer period of 192 hr and the complete NO_2_^−^ reduction process was followed over time, and growth and nitrogen compound concentrations were monitored at several time points to study the mechanism of N_2_O production.

### 2.3 Analytical procedures

Samples of 1 ml were taken from cultures through the rubber septum of serum vials with sterile syringes for growth determination and colorimetric determination of NH_4_^+^, NO_3_^−^, and NO_2_^−^. Growth was determined by measuring the optical density OD_600_ of 100 μl sample in duplicate in microtiter plates and standardized to 1 cm path length using PathCheck Sensor of the spectrophotometer (Molecular Devices, Spectramax plus 384, USA). Samples left were centrifuged at 17,949*g* for 2 min to remove the cells, and supernatants were kept frozen at ‐20°C until colorimetric determination. NH_4_^+^ concentration was determined with the salicylate‐nitroprusside method (absorption at a wavelength of 650 nm) (Baethgen & Alley, 1989), and NO_2_^−^ and NO_3_^−^ concentrations were determined with Griess reaction (Griess, 1879) and Griess reaction with cadmium (Cataldo, Haroon, Schrader, & Youngs, 1975; Navarro‐Gonzalvez, Garcıa‐Benayas, & Arenas, 1998), respectively. For endpoint measurements, NH_4_^+^ production was corrected per strain for the amount of NH_4_^+^ assimilated based on OD_600_ values obtained. Standard curves covered ranges suitable for the tested media and were strictly linear with an R_2_ of 0.99. For determination of N_2_O, 1 ml sample of the headspace of serum vials was taken with sterile syringes and was injected into a gas chromatograph (Compact GC with EZChrom Elite Software, Interscience, Netherlands, 2012, column molsieve 5A 7*0.32 mm and Rt‐Q Bond 3*0.32 mm). N_2_O concentrations were corrected for pressure and solubility based on Henry's law. Henry's constant for N_2_O is 0.025 mol/L/atm at 25°C.

Statistical differences in end product concentration (OD_600_, NO_3_^−^/NO_2_^−^/NH_4_^+^ concentration, N_2_O production) and ratios of N‐NH_4_^+^ production to N–N_2_O production (indicating NO_3_^−^ partitioning to NH_4_^+^ and N_2_O) in the tests of different environmental drivers were processed using factorial ANOVA and least significant difference post hoc testing in IBM SPSS 23 or the nonparametric Kruskal–Wallis *H* test.

## 3 RESULTS AND DISCUSSION

### 3.1 NO_2_^−^ reduction ability

**Figure 1:**
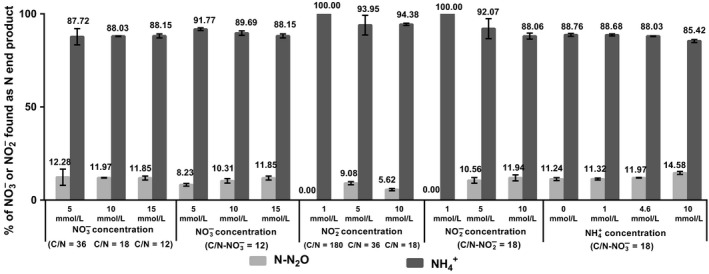
Production of nitrous compounds by *Bacillus paralicheniformis* LMG 6934 in different mineral media after 72‐hr anaerobic incubation. Percentages of end products of anaerobic NO_3_^−^/NO_2_^−^ reduction in mineral medium with increasing NO_3_^−^ concentration under variable C/N‐NO_3_^−^ ratio (*n* = 2 for C/N ratio of 36); with increasing NO_3_^−^ concentration under fixed C/N‐NO_3_^−^ ratio of 12 (for 15 mmol/L NO_3_^−^, it is the same experiment as above, the same data used twice for analysis); with increasing NO_2_^−^ concentration under variable C/N‐NO_2_^−^ ratios; with increasing NO_2_^−^ concentration under fixed C/N‐NO_2_^−^ ratio of 18; with increasing NH_4_^+^ concentration under fixed C/N‐NO_3_^−^ ratio of 18. Error bars represent standard deviation (*n* = 3 if not stated otherwise). Measured concentrations of NH_4_^+^ were corrected for loss through assimilation

Already three decades ago, it was suggested that N_2_O production during DNRA originates from detoxification of accumulated NO_2_^−^ (Bleakley & Tiedje, 1982; Smith, 1983). Our previous study demonstrated that *B. paralicheniformis* LMG 6934 had a high NO_2_^−^ tolerance of 10 mmol/L and could efficiently perform DNRA by reducing all intermediary NO_2_^−^ to NH_4_^+^ and N_2_O (Sun et al., 2016), while *B. paralicheniformis* LMG 7559 showed a NO_2_^−^ tolerance of 6.29 ± 0.39 mmol/L, and both LMG 7559 and *B. licheniformis* LMG 17339 had residual NO_2_^−^ (2.76 mmol/L ± 0.57 mmol/L, 4.88 mmol/L ± 0.60 mmol/L) after 72‐hr incubation probably due to their lower tolerance to the toxic effect of NO_2_^−^. Less N_2_O was produced by LMG 6934 than by LMG 7559 and LMG 17339, and less NO_3_^−^ partitioning to N_2_O was observed as well ([N‐NH_4_^+^]/[N–N_2_O] of 4.24 ± 0.29 vs 1.49 ± 0.82, 0.71 ± 0.09, respectively) (Sun et al., 2016 and unpublished data therein). To uncover factors affecting N_2_O production during DNRA, here, NO_2_^−^ reduction was anaerobically tested in LMG 6934 at concentrations of 1 mmol/L, 5 mmol/L, and 10 mmol/L under variable C/N‐NO_2_^−^ ratios of 180, 36, and 18 and fixed C/N‐NO_2_^−^ ratios of 18. After 72‐hr incubation, growth was observed under all NO_2_^−^ concentrations tested, with all NO_2_^−^ converted to NH_4_^+^ or N_2_O, thus confirming its high tolerance to NO_2_^−^ (Table 1; Figure 1). Indeed, compared with other DNRA strains (Sun et al., 2016) belonging to *Bacillus* sp. and *Citrobacter* sp. (Stremińska et al., 2012), *B. licheniformis* (Konohana et al., 1993), and *Pseudomonas stutzeri* D6 (Yang, Wang, & Zhou, 2012), LMG 6934 showed a high NO_2_^−^ reduction ability, with up to 10 mmol/L of initial NO_2_^−^ consumed. Furthermore, up to 15 mmol/L NO_3_^−^ was converted to NH_4_^+^ (>85%) and N_2_O (<15%) with no residual NO_2_^−^ at the end of the experiment. The high NO_2_^−^ reduction ability observed in our tests with high NO_3_^−^ or NO_2_^−^ concentration might partly be due to increased NirB activity (Wang & Gunsalus, 2000).

**Table 1:** Overview of growth tests of *Bacillus paralicheniformis* LMG 6934

Media supplements	C/N‐NO_*x*_^−^	∆OD_600_	Concentration (mmol/L)		
			NO_3_^−^ or NO_2_^−^ consumed	NH_4_^+^ produced	N_2_O produced
5 mmol/L NO_3_^−^	36	0.60^aA^ (0.10)	5.23^aA^ (0.15)	4.80^aA^ (0.27)	0.33^aA^ (0.12) *
10 mmol/L NO_3_^−^ #	18	0.71^aAB^ (0.20)	9.87^bA^ (0.43)	8.69 A^b^ (0.36)	0.59^bA^ (0.03)
15 mmol/L NO_3_^−^ ##	12	0.76^a^ (0.09)	14.67^c^ (1.13)	12.94^c^ (1.15)	0.87^c^ (0.02)
5 mmol/L NO_3_^−^	12	0.22^aB^ (0.03)	4.91^aA^ (0.21)	4.50^aA^ (0.23)	0.20^aA^ (0.01)
10 mmol/L NO_3_^−^	12	0.50^bA^ (0.05)	9.55^bA^ (1.13)	8.57^bA^ (1.11)	0.49^bB^ (0.01)
15 mmol/L NO_3_^−^ ##	12	0.76^c^ (0.09)	14.67^c^ (1.13)	12.94^c^ (1.15)	0.87^c^ (0.02)
1 mmol/L NO_2_^−^	180	0.35^a^ (0.02)	1.17^a^ (0.01)	1.17^a^ (0.01)	0^a^ (0.00)
5 mmol/L NO_2_^−^	36	0.51^bA^(0.02)	6.19^bB^ (0.17)	5.71^bB^ (0.15)	0.19^abA^ (0.16)
10 mmol/L NO_2_^−^	18	0.66^cA^ (0.03)	13.76^cB^ (0.97)	12.99^cB^ (0.99)	0.39^bC^ (0.01)
1 mmol/L NO_2_^−^	18	0.22^a^ (0.01)	0.99^a^ (0.01)	0.99^a^ (0.01)	0^a^ (0.00)
5 mmol/L NO_2_^−^	18	0.52^bA^ (0.06)	4.87^bA^ (0.06)	4.35^bA^ (0.07)	0.26^bA^ (0.04)
10 mmol/L NO_2_^−^	18	0.95^cBC^ (0.10)	9.57^cA^ (0.17)	8.53^cA^ (0.16)	0.55^cABC^ (0.08)
0 mmol/L NH_4_^+^	18	0.67^aAB^ (0.08)	10.32^aAB^ (1.34)	9.16^aA^ (1.26)	0.58^aA^ (0.04)
1 mmol/L NH_4_^+^	18	0.82^aB^ (0.02)	10.95^aAB^ (0.18)	9.71^aA^ (0.20)	0.62^aA^ (0.02)
4.6 mmol/L NH_4_^+^ #	18	0.71^aAB^ (0.20)	9.87^aA^ (0.43)	8.69^aA^ (0.36)	0.59^aA^ (0.03)
10 mmol/L NH_4_^+^	18	0.87^aB^ (0.03)	8.99^aA^ (0.99)	7.68^aA^ (0.91)	0.65^aA^ (0.04)

Growth (∆OD_600_), electron acceptors (NO_3_^−^ or NO_2_^−^) consumption, NH_4_^+^ production (measured concentrations of NH_4_^+^ corrected for loss through assimilation), and N_2_O production after 72‐hr incubation under different media composition are shown. All NO_3_^−^ added was consumed by the end of the experiment. Standard deviations are given between brackets (*n* = 3 if not stated otherwise). Statistics were determined via one‐way ANOVA or nonparametric tests accordingly. Significant differences (*p *<* *.05) of each parameter (OD_600_, NO_3_^−^ or NO_2_^−^ consumption, NH_4_^+^, and N_2_O production) within the same experiment (five experiments: (i) NO_3_^−^ concentration test under variable C/N^−^ NO_3_^−^ ratio, (ii) NO_3_^−^ concentration test under fixed C/N− NO_3_^−^ ratio, (iii) NO_2_^−^ concentration test under variable C/N^−^ NO_3_^−^ ratio, (iv) NO_2_^−^ concentration test under fixed C/N^–^ NO_3_^−^ ratio, and (v) NH_4_^+^ concentration test (with initial 10 mmol/L NO_3_^−^)) are displayed as different lowercase letters (combined lower letters are used to indicate nonsignificance for multiple variables). Significant differences in each parameter between four different experiments when 5 mmol/L NO_3_^−^/NO_2_^−^ or 10 mmol/L NO_3_^−^/NO_2_^−^ supplied is displayed as capital letters.

^*^ *n* = 2.

^#^or ^##^indicates data from the same test analyzed twice in different experiment interpretation.

### 3.2 Influence of NO_**3**_^**−**^ and NO_**2**_^**−**^ concentration on N_2_O production

Anaerobic growth experiments with 5, 10, and 15 mmol/L NO_3_^−^ under variable C/N‐NO_3_^−^ ratios of 36, 18, and 12 after 72‐hr incubation revealed that NO_3_^−^ or intermediate NO_2_^−^ was completely converted to N_2_O or NH_4_^+^ for all conditions tested and growth ceased and sporulation started due to either NO_3_^−^ limitation for respiration or carbon source (glucose) limitation for fermentation. Growth (∆OD_600_) (including sporulation), consumption of NO_3_^−^, production of NO_v_ and NH_4_^+^ are summarized in Table 1. Percentages of NO_3_^−^ or NO_2_^−^ converted to N_2_O or NH_4_^+^ under different conditions are shown in Figure 1. Percentage of NO_3_^−^ recovery as N_2_O and growth (∆OD_600_) under 10 mmol/L NO_3_^−^ condition agreed with previous observations (Sun et al., 2016).

**Figure 2:**
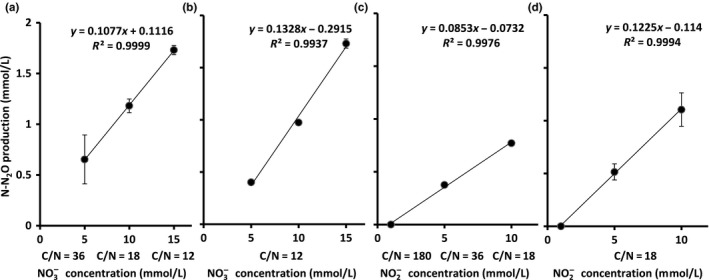
N–N_2_O production by *B. paralicheniformis* LMG 6934 in different mineral media after 72‐hr anaerobic incubation. Media tested are supplemented with the following: (a) increased NO_3_^−^ concentration under variable C/N‐NO_3_^−^ ratio of 36 (*n* = 2), 18, and 12; (b) increased NO_3_^−^ concentration under fixed C/N‐NO_3_^−^ ratio of 12; (c) increased NO_2_^−^ concentration under variable C/N‐NO_2_^−^ ratio of 180, 36, and 18; (d) increased NO_2_^−^ concentration under fixed C/N‐NO_2_^−^ ratio of 18. Error bars represent standard deviation (*n* = 3 if not stated otherwise). Trend line equations and R‐squared value are given

**Figure 3:**
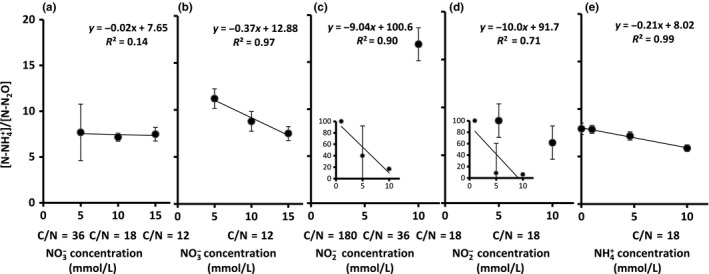
Ratio of N‐NH_4_^+^ production to N–N_2_O production by *B. paralicheniformis* LMG 6934 after 72‐hr anaerobic incubation in mineral media. Mineral medium supplemented with the following: (a) increasing NO_3_^−^ concentration under variable C/N‐ NO_3_^−^ ratio of 36 (*n* = 2), 18, and 12; (b) increasing NO_3_^−^ concentration under fixed C/N‐NO_3_^−^ ratio of 12; (c) increasing NO_2_^−^ concentration under variable C/N‐NO_2_^−^ ratio of 180, 36, and 18; (d) increasing NO_2_^−^ concentration under fixed C/N‐ NO_2_^−^ ratio of 18; (e) increasing NH_4_^+^ concentration under fixed C/N‐ NO_3_^−^ ratio of 18. Error bars represent standard deviation (*n* = 3 if not stated otherwise). The inserted figure in panel C and panel D is the complete figure of the test with a [N–NH_4_^+^]/[N–N_2_O] range from 0 to 100. Trend line equations and *R*‐squared value are given

With a constant 30 mmol/L glucose and variable C/N‐NO_3_^−^ ratios of 36, 18, and 12, the rising NO_3_^−^ concentration had an influence on N_2_O production (*p* = .0018) and NH_4_^+^ production (*p* = .000027), with higher NO_3_^−^ concentrations leading to production of more NH_4_^+^ and more N_2_O (Table 1; Figure 2a). Different NO_3_^−^ concentrations had no significant influence on NO_3_^−^ partitioning ([N–NH_4_^+^]/[N–N_2_O]) (*p* = .417) (Figure 3a). Growth did not significantly increase with NO_3_^−^ concentration (*p* = .287) (Figure 3a), and this may because excess glucose (initial 30 mmol/L) supports fermentation and sporulation. Smith (1981) showed that, in *Citrobacter,* higher C/N‐NO_3_^−^ ratios with constant NO_3_^−^ concentration favor NO_3_^−^ partitioning to N_2_O. In our study, the opposite was apparently found: A higher C/N‐NO_v_ ratio led to less N_2_O produced. However, the higher C/N‐NO_3_^−^ ratios here were created by lowering NO_3_^−^ concentration with glucose at 30 mmol/L. We hypothesize that lower NO_3_^−^ concentration would lead to lower NO_2_^−^ concentration resulting in a lower toxic effect and less need for its reduction to nontoxic N_2_O. To confirm that a rising NO_3_^−^ concentration and exclude the influence of C/N‐NO_3_^−^ ratio, which might be strain‐dependent (Stremińska et al., 2012), the same experiment was repeated under fixed C/N‐NO_3_^−^ ratio of 12. Again, after 72‐hr anaerobic incubation, all NO_3_^−^ or NO_2_^−^ was completely converted to N_2_O or NH_4_^+^ without any residual NO_2_^−^ left for all conditions tested. As expected, growth increased with a rising NO_3_^−^ concentration under fixed C/N‐NO_3_^−^ ratio (*p* = .000128) and was supported by fermentation of glucose and NO_3_^−^ respiration. NH_4_^+^ production (*p* = .000101) and N_2_O production (*p* = 4.95 × 10^−9^) showed a positive correlation with the rising NO_3_^−^ concentration (Table 1; Figure 2b). In addition, increased NO_v_ concentration from 5 to 10 mmol/L promoted NO_3_^−^ partitioning to N_2_O and negatively impacted its partitioning to NH_4_^+^ (*p* = .008) (Figure 3b), but this effect was statistically not significant when increasing from 10 to 15 mmol/L NO_3_^−^ (*p* = .155).

In contrast to a rising NO_3_^−^ concentration under variable C/N‐NO_3_^−^ ratios, a rising NO_2_^−^ concentration under variable C/N‐NO_2_^−^ ratio did show a positive effect on NH_4_^+^ production (*p* = .027) and N_2_O production (*p *=* *.034) and resulted in an increasing growth (*p *=* *.000017) supported by fermentation and/or respiration as stated above. However, why this excess glucose did not result in similar growth by fermentation as it did in NO_3_^−^ concentration tests is unclear. As expected, with more NO_2_^−^ consumed in the media, more NH_4_^+^ and N_2_O were produced, resulting in more cell growth (Table 1; Figure 2c). In addition, increase in NO_2_^−^ concentration had a significantly positive influence on NO_2_^−^ partitioning to N_2_O but the significance was only shown between 1 mmol/L and 10 mmol/L NO_2_^−^ (*p *=* *.00028) (Figure 3c), which is also the case for the amount of N_2_O produced (Table 1).

**Figure 4:**
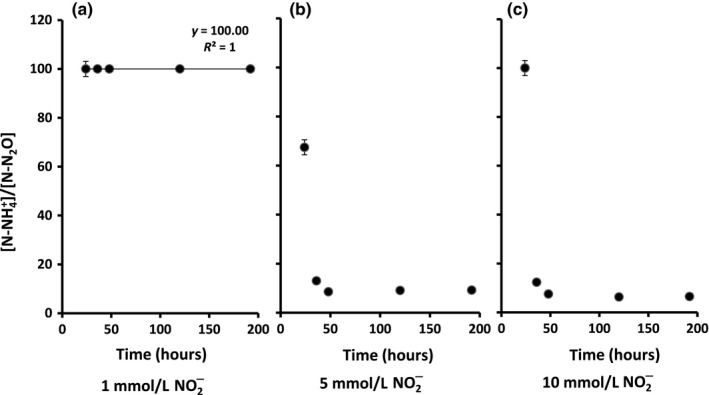
Ratio of N–NH_4_^+^ production to N–N_2_O production during 192 hr of anaerobic incubation of *B. paralicheniformis* LMG 6934 in mineral medium supplemented with NO_2_^−^ under fixed C/N‐ NO_2_^−^ ratio of 18: (a) 1 mmol/L NO_2_^−^ added; (b) 5 mmol/L NO_2_^−^ added; and (c) 10 mmol/L NO_2_^−^ added

Similarly, increasing NO_2_^−^ concentration under fixed C/N‐NO_2_^−^ ratio of 18 also showed a positive effect on growth (*p *=* *.000049), NH_4_^+^ production (*p *=* *1.9996E−8), and N_2_O production (*p *=* *.000033) (Table 1; Figure 2d). Likewise, rising NO_2_^‐^ concentration had a significantly positive influence on NO_2_^−^ partitioning to N_2_O, but the significance was only shown between 1 mmol/L and 5 mmol/L or 10 mmol/L NO_2_^−^ (*p *=* *7.5916E−11) (Figure 3d). To further study the conditions affecting N_2_O production during DNRA, growth was monitored over a 192‐hr incubation period. As expected, NH_4_^+^ was produced during incubation, accompanied by N_2_O production and NO_2_^−^ partitioning to N_2_O at first increased, becoming stable after 48 hr (Figure 4).

In summary, a linear but nonstoichiometric correlation was observed for the first time between NO_3_^−^ or NO_2_^−^ concentration and N_2_O production (Figure 2), which may be useful for further studies of N_2_O production calculation or interpretation of its regulation. In addition, increasing NO_3_^−^ concentration under fixed C/N‐NO_3_^−^ ratio but not under variable C/N‐NO_3_^−^ ratios and increasing NO_2_^−^ concentration under variable as well as fixed C/N‐NO_2_^−^ ratios significantly increased NO_3_^−^ partitioning to N_2_O in *B*. *paralicheniformis* LMG 6934 (Figure 3). The latter may be a direct effect of NO_2_^−^, probably by action of NirB, while NO_3_^−^ may work through a combined effect of C/N‐NO_3_^−^ ratio and NO_3_^−^ concentration. Higher NO_3_^−^ concentration under fixed C/N‐NO_3_^−^ ratio promotes NO_3_^−^ partitioning to N_2_O, and this agrees with physiological data of a previous study (Smith, 1981). It indeed makes sense that, under higher NO_3_^−^ concentration, more NO_2_^−^ transiently accumulates and therefore needs to be detoxified, leading to a higher proportion of NO_3_^−^ to N_2_O. This agrees with the observation in NO_2_^−^ batch tests. Non‐negligibly, the C/N‐NO_3_^−^ referred to was the initial ratio. The C/N‐NO_3_^−^ ratio varied during the batch incubation tests. Constant C/N‐NO_3_^−^ in a chemostat setup is suggested for further study.

### 3.3 Influence of NH_4_^+^ concentration on N_2_O production

It is known that NH_4_^+^ can repress NO_3_^−^ assimilation causing NO_2_^−^ to accumulate (Schreier et al., 1989; Stouthamer, 1976); however, it does not inhibit nitrate reduction for dissimilation toward NH_4_^+^ (Konohana et al., 1993). Here, we tested its effect on N_2_O production and used NH_4_^+^ concentrations of 0 mmol/L, 1 mmol/L, 4.6 mmol/L (standard), and 10 mmol/L in the presence of 10 mmol/L NO_3_^−^ under a fixed C/N‐NO_3_^−^ ratio of 18. After 72‐hr incubation, growth was obtained under all NH_4_^+^ concentrations, even without NH_4_^+^ added (Table 1; Figure 1). All NO_3_^−^ was converted to NH_4_^+^ or N_2_O, with some samples reaching up to approx. 10 mmol/L NH_4_^+^ produced (Table 1). There was no statistically significant effect of NH_4_^+^ concentration on growth (*p *=* *.12) as expected, and similar results were observed for NH_4_^+^ production (*p *=* *.12) or N_2_O production (*p *=* *.11), again confirming that LMG 6934 is a vigorous ammonifier able to produce and take up sufficient NH_4_^+^ for growth. However, there was a significant effect of NH_4_^+^ on NO_3_^−^ partitioning to N_2_O but only in medium with the highest NH_4_^+^ concentration (10 mmol/L) compared with media with lower NH_4_^+^ concentration (*p *=* *.000932) (Figure 3e). This observation requires further confirmation with higher NH_4_^+^ concentrations, and this mechanism behind this effect requires in‐depth study.

Thus, anaerobic growth was not repressed by NH_4_^+^ (starting from 10 mmol/L initial NH_4_^+^, an NH_4_^+^ concentration as high as 18.47 ± 0.10 mmol/L was measured after incubation), which is in agreement with previous studies on *Bacillus* sp. and *Citrobacter* sp. (Smith & Zimmerman, 1981). Almost no difference in growth was obtained under different NH_4_^+^ concentrations. Similar observations were described with *B. licheniformis* No. 40‐2, a strain isolated from a hot spring but under aerobic conditions (Konohana et al., 1993).

### 3.4 Ecological relevance and future perspectives

Here, we demonstrated that indeed NO_3_^−^ as well as NO_2_^−^ concentration shows a linear correlation with N_2_O production and increasing concentrations lead to more partitioning to N_2_O which may be a direct result of NO_2_^−^ detoxification. This linear correlation is media‐dependent and may be strain‐dependent, as was found in our previous study when comparing three *Bacillus* strains in different media conditions (Sun et al., 2016). The underlying mechanisms, however, remain elusive. Further studies are required to assess whether these effects apply for other DNRA strains and under field conditions. Such information may in future contribute to the estimation of environmental N_2_O emissions based on in situ measurements of environmental parameters. Furthermore, we also observed that higher NH_4_^+^ concentration could lead to more NO_3_^−^ partitioning to N_2_O. Canonical NO reductase (Nor) is widespread among denitrifiers and nondenitrifiers and efficient for NO reduction to N_2_O. The genome of strain LMG 6934 encodes for quinol‐dependent NO reductase (qNor) as well as Hmp (Sun et al., 2016). Hmp, however, has not been fully proved to be physiologically relevant as protection from nitrosative stress (Torres et al., 2016). Therefore, as there was no growth defect caused by NO toxicity under the conditions tested, it can be hypothesized that qNor rather than Hmp may be a significant source of N_2_O in LMG 6934. However, it still remains unclear whether NO generation is by NarG, NirBD, or both of them.

This study contributed to characterization of DNRA performance under different environmental drivers, including increasing NO_3_^−^, NO_2_^−^, and NH_4_^+^. Although we used relatively high concentrations of NO_3_^−^ or NO_2_^−^, they are still relevant as comparable concentrations can exist in the environment (Reisenauer, 1966; Wolt, 1994), for example during fertilization events of agricultural land (Dechorgnat et al., 2011). We realize that the N_2_O production during ammonification might be considered negligible compared to that during canonical denitrification, especially when considering LMG 6934 is highly tolerant to NO_2_^−^. Nevertheless, ammonifiers are widely distributed in the environment and DNRA is considered the preferred NO_3_^−^ reduction process in agricultural soils as it retains N in the system (Mania et al., 2014). Therefore, future N_2_O mitigation strategies promoting DNRA need to consider the potential concomitant N_2_O production. In this respect, *B. paralicheniformis* LMG 6934, which under laboratory conditions produces less N_2_O than some other DNRA bacteria (Sun et al., 2016), is an interesting strain. It was originally isolated from garden soil, showing nonfastidious growth and is nonpathogenic and may thus be a good candidate for application in agricultural fields, to promote DNRA over denitrification. This would favor nitrogen retention, increasing efficiency of nitrogen fertilizer applied and, to a certain degree, reducing N_2_O emission from the soil.

## CONFLICT OF INTEREST

The authors declare that they have no conflict of interest.
